# Correlations between the structure and the vibrational spectrum of the phosphate group. Implications for the analysis of an important functional group in phosphoproteins†

**DOI:** 10.1039/c9ra10366j

**Published:** 2020-01-29

**Authors:** Pontus Pettersson, Andreas Barth

**Affiliations:** Department of Biochemistry and Biophysics, Arrhenius Laboratories, Stockholm University 10691 Stockholm Sweden barth@dbb.su.se

## Abstract

Density functional theory calculations were used to establish correlations between the structure and the vibrational spectrum of the phosphate group in model compounds for phosphorylated amino acids. The model compounds were acetyl phosphate, methyl phosphate, and *p*-tolyl phosphate, which represented the phosphorylated amino acids aspartyl phosphate, serine or threonine phosphate, and tyrosine phosphate, respectively. The compounds were placed in different environments consisting of one or several HF or H_2_O molecules, which modeled interactions of phosphorylated amino acids in the protein environment. The calculations were performed with the B3LYP functional and the 6-311++G(3df, 3pd) basis set. In general, the wavenumbers (or frequencies) of the stretching vibrations of the terminal P–O bonds correlated better with bond lengths of the phosphate group than with its bond angles. The best correlations were obtained with the shortest and the mean terminal P–O bond lengths with standard deviations from the trend line of only 0.2 pm. Other useful correlations were observed with the bond length difference between the shortest and longest terminal P–O bond and with the bond length of the bridging P–O bond.

## Abstract 2

Vibrational frequencies of phosphate are sensitive to bond length changes on the sub-picometer scale.

## Graphical abstract

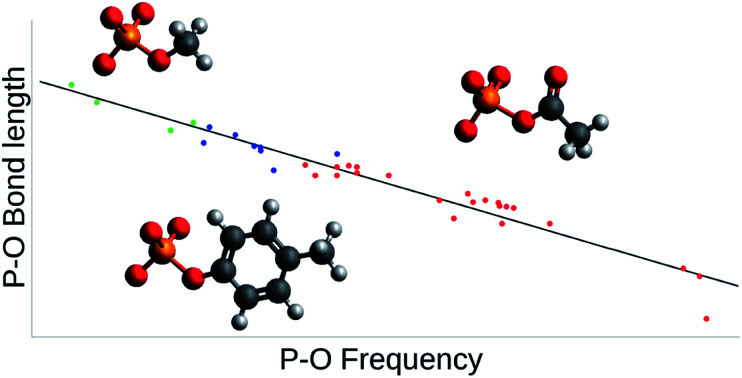

## 1 Introduction

Phosphorylation of amino acid side chains in proteins controls a large number of biological processes.^1,2^ Important parameters for these regulation mechanisms are the rates of phosphorylation and dephosphorylation, which are determined by interactions between phosphate group and its vicinity. These interactions will reflect in subtle changes in the bond lengths and the bond angles of the phosphate group, which modify the bond strength of the scissile P–O bond and thus its reactivity. Some of the bond distortions relevant for the transition state of a reaction may already be present in the ground state,^3–6^ which makes them assessable by structural methods.

While the interactions between the phosphate group and its environment can be visualized by *e.g.* X-ray crystallography, their effect on bond strengths is more difficult to measure because of the experimental uncertainty in the determination of bond lengths (0.1 Å for a 1.2 Å resolution structure).^7^ On the other hand, such information can be obtained from vibrational spectroscopy, as discussed in more detail previously,^8^ due to the tight correlation between vibrational spectrum and bond strengths. The experimental approach often uses a combination of difference spectroscopy^9–12^ and isotope labelling^13–17^ to identify the few phosphate vibrations in a crowded vibrational spectrum.

With this background it seems desirable to explore the structural information that is available from the phosphate vibrations and to extend previous work on this topic that did not consider interactions between phosphate group and its environment.^18^ We have earlier studied models for phosphorylated amino acids while interacting with HF or H_2_O molecules.^19^ The aim then was to understand how different electrostatic interactions in an enzyme can affect the reaction mechanism. Here, we present results from density functional theory (DFT) calculations made in order to correlate experimentally observable wavenumbers (which are proportional to vibrational frequencies) with phosphate structure. We focus on the P–O_T_ stretching vibrations as these are best accessible in experiments because they are strong absorbers of infrared radiation and are found above 900 cm^−1^ where the absorption from water and from the commonly used CaF_2_ windows is comparably low.

Our interest in this investigation originated from the aim to determine the bond strength of the P–O bond that bridges the phosphate group and Asp351 of the phosphorylated Ca^2+^-ATPase (SERCA1a). We wanted to find out whether the protein environment weakens the bond before it is cleaved upon phosphoenzyme hydrolysis. For this purpose, we identified the phosphate vibrations by an isotope exchange experiment.^16^ These were first evaluated by empirical correlations^16^ and later by DFT calculations^8^ which led to conflicting results regarding the effect of the enzyme environment on the bond strength of the bridging P–O bond. Here, we reproduce the general trend of the empirical correlations in DFT calculations but show also that the scatter around the trend line is large enough to cause the erroneous interpretation of our first publication.^16^ Preliminary accounts of this work have been published in the PhD thesis of Maria Rudbeck^20^ and in the Bachelor's thesis of Pontus Pettersson.^21^

## 2 Material and methods

### 2.1 Structural models

**Fig. 1:**
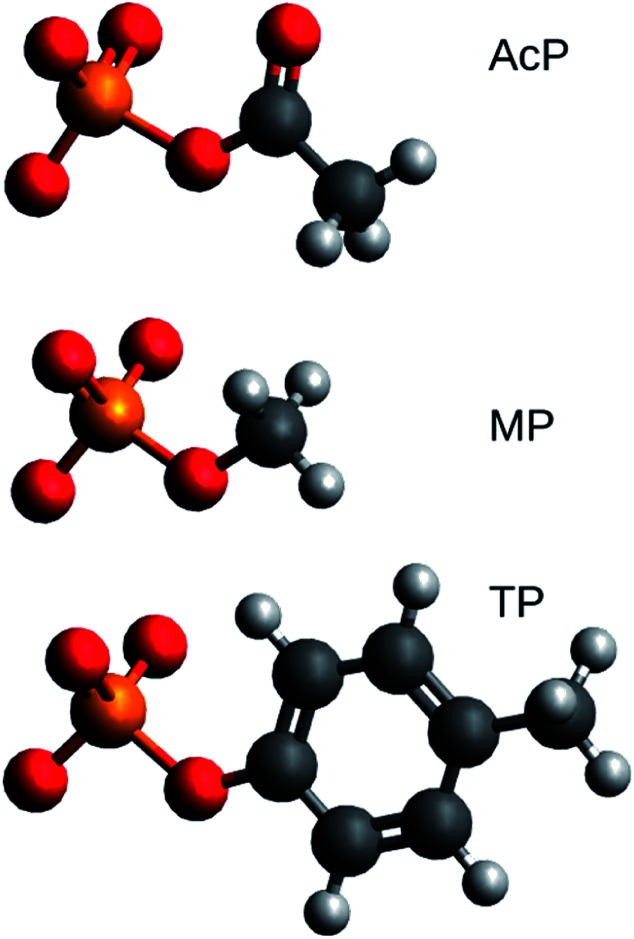
Structures of acetyl phosphate (AcP), methyl phosphate (MP), and *p*-tolyl phosphate (TP). Carbon atoms are dark grey, hydrogen atoms light grey, oxygen atoms red and the phosphorus atom orange. The figure was generated with Avogadro.

Structural models of acetyl phosphate, methyl phosphate, and *p*-tolyl phosphate were studied. Their chemical structures are shown in Fig. 1. They represent the phosphorylated amino acids aspartyl phosphate, serine or threonine phosphate, and tyrosine phosphate, respectively.

The molecules were placed in (generally asymmetric) environments consisting of zero to six H_2_O molecules or zero to three HF molecules resulting in a total number of models of 34. HF has only a single hydrogen bond donor, whereas H_2_O has two, which makes it more straightforward to model an intended interaction with HF. Also, the HF bond is very polar, which makes it possible to model very strong electrostatic interactions. We have previously shown that interactions with HF and water follow the same trend lines when structural distortions of phosphate groups are correlated.^19^

The following interactions were considered: hydrogen-bonding to the terminal oxygens (O_T_⋯H), hydrogen-bonding to the bridging oxygen of the phosphate (O_B_⋯H), a nucleophilic attack towards the phosphorus (P⋯F or P⋯O), and hydrogen bonding to the carbonyl oxygen (C=O⋯H) for acetyl phosphate. In a number of models, some distances were constrained, which is detailed in the following. For 10 of 34 models, O_T_⋯H distances between the interacting molecules and the phosphorylated molecules were fixed to distances between 1.5 and 2.5 Å. The same was done for the O_B_⋯H distance in 12 of the models and for the C=O⋯H distance in 5 of the models. In 4 models, the P⋯F or P⋯O distance was constrained to 2.5 Å. For nearly half of the models with HF molecules (6 out of 14), the HF length was constrained either to 0.94 Å (5 models) or to 0.93 Å (one model), which was smaller than the HF bond length in unconstrained calculations. For one model, also the O⋯HF angle was constrained. The models are described in more detail in ESI.†

Some of the acetyl phosphate models were directly related to our work on the Ca^2+^-ATPase.^8^ They are named AcP-E2P models 1 to 3 as in the previous work (see the ESI† of our 2015 article for the structure of the models). AcP-E2P model 1 reproduced the wavenumbers that were experimentally found for the E2P phosphoenzyme (1194 and 1137 cm^−1^).

### 2.2 DFT calculations

All geometry optimizations of the structural models were performed with the Gaussian03 program.^22^ The geometries were fully optimized, with a few constraints as described above, using DFT with the B3LYP functional^23,24^ and the 6-311++G(3df, 3pd) basis set.

All wavenumber calculations were performed on the optimized structures using Gaussian03. No scaling factor was applied to the wavenumber calculations since our basis set calculates P–O wavenumbers which are very close to experimental results:^25^ for the two PO_3_^2−^-containing molecules in the studied set of molecules, the calculated wavenumbers of the asymmetric P–O stretching vibrations differed by only 3.3 cm^−1^ from experimental results (averaged absolute value of the deviations). One of these molecules was acetyl phosphate. The wavenumbers calculated with the 6-311++G(3df, 3pd) basis set can therefore directly be compared to experimental results.

Anharmonicity is expected to influence the P–O_T_ stretching vibrations only by a few cm^−1^,^26,27^ and was not considered in our calculations. In spite of the neglect of anharmonicity in our calculations, the agreement with experiment is excellent as stated above. This is probably caused by a cancellation of errors.

Solvent effects were included, in both the optimization and the frequency calculations using the self-consistent polarization model (PCM). More specifically, the conductor-like screening model (CPCM)^28,29^ was used with the default settings for water (dielectric constant *ε* = 78.39) or *ε* = 4, which is commonly used when modeling a protein environment.

### 2.3 Evaluation of the DFT data

An Octave program was written to evaluate the Gaussian output files. The program scanned the files for the relevant data, for example atom positions, structural constraints, wavenumbers, infrared intensities, displacement vectors of the atoms for the different normal modes, and dielectric constant. The selected data were used to identify the atoms of the phosphate group and the associated carbon atom based on the distances to the only phosphorus atom in the models. Atom positions were used to calculate bond lengths and angles within the group and distances between terminal oxygens and ligands. The number of carbon, oxygen and fluorine atoms was used by the Octave program to identify the phosphorylated molecule and the identity and number of ligands.

To identify the symmetric and asymmetric stretching vibrations of the terminal oxygens of the phosphate group, the following procedure was used. The difference between the displacement vector of each terminal oxygen (O_T_) and the displacement vector of the phosphorus was calculated for each normal mode in the wavenumber interval of the P–O_T_ stretching vibrations. Each resultant P–O_T_ displacement vector was then projected on a unit vector from the phosphorus atom to the corresponding O_T_ creating a scalar that reflected how much of the vibrational motion was related to stretching of the P–O_T_ bond. The signs of the three projections were used to distinguish symmetric and asymmetric stretching vibrations. According to Hooke's law, the energy of bond stretching is proportional to the squared projection. Accordingly, the projections for the three P–O_T_ bonds were squared and summed to obtain a value that is proportional to the energy contribution of the P–O_T_ stretching vibrations to the normal mode considered. The two modes with the highest contributions and with projections with different signs were assigned to the two asymmetric stretching vibrations of the phosphate group.

Identifying the symmetric PO_3_^2−^ stretching vibration was more complex: in most cases there was only one symmetric stretching vibration with a large energy contribution from the P–O_T_ stretching vibrations and the assignment was straightforward. However, for some models there were several normal modes with symmetric P–O_T_ stretching vibrations. In these cases, those two with the highest infrared intensities were compared. Often, the normal modes also involved the motion of the bridging oxygen O_B_. Its motion was then further analyzed to identify the nature of the two normal modes: the projection of the P–O_B_ displacement vector on the P–O_B_ unit vector was squared and compared with the energy contribution of P–O_T_ stretching vibrations to the normal mode. Only vibrations with P–O_T_ energy contributions at least as large as the P–O_B_ energy contribution were kept for further analysis. If there were still two symmetric normal modes left, their P–O_T_ energy contributions were compared. In 32 out of 34 cases, the P–O_T_ energy contribution to one normal mode was less than half the contribution to the other mode. In these cases the normal mode with the smaller energy contribution was discarded. In two other cases, a new average of the two wavenumbers was created by weighting the wavenumbers with infrared intensities of the respective normal modes. The average is an approximation of the experimentally observed band position which is determined by the overlap of both bands. For both models, a vibration with high intensity was calculated near 950 cm^−1^ and one with ten-fold lower intensity near 970 cm^−1^, so that the weighted average was dominated by the 950 cm^−1^ vibration.

Many models were manually checked for faulty selection of vibrations but no error was found. The wavenumbers of the P–O_T_ stretching vibrations as well as structural parameters of the phosphate group are compiled in ESI.†

### 2.4 Correlations between structural parameters and vibrational properties

Structural parameters of the phosphate group were plotted against the wavenumbers of the asymmetric P–O_T_ stretching vibrations *ṽ*_as_(P–O_T_), their difference Δ*ṽ*_as_(P–O_T_), and the fundamental wavenumber *ṽ*_f_. The latter was defined as *ṽ*_f_ = [(*ṽ*_as1_^2^ + *ṽ*_as2_^2^ + *ṽ*_s_^2^)/3]^1/2^, where *ṽ*_as1_ and *ṽ*_as2_ are the wavenumbers of the two asymmetric stretching vibrations and *ṽ*_s_ the wavenumber of the symmetric stretching vibration. This definition is an extension of the previous definition^18^ to the asymmetric phosphate environments in our structural models. Straight lines were fitted to the data in which the sum of the squared deviations between the DFT values of the structural parameters and the values predicted by the trend lines were minimized.

### 2.5 Bond valence

The bond valence model^30,31^ has its origin in an ionic model of bonds between atoms but has been found applicable also for polar covalent bonds. It states that the sum of the bond valences of the bonds of an atom equals the atomic valence of the atom. The bond valence of a bond depends on the bond length *L* and two equations have been put forward to relate the two quantities.

1 *S* = (*L*_1_/*L*)^*N*^

2 *S* = exp[(*L*_1_ − *L*)/*B*]

*S* is the experimental bond valence in valence units, *L* is the bond length and *L*_1_ is the bond length for *S* = 1. *L*_1_, *N*, and *B* are experimentally determined constants of which the former two depend on the type of bond, whereas the latter is often regarded as a universal constant (but see the discussion in Brown, 2009).^30^Eqn (1) has been proposed earlier,^32^ but eqn (2) (ref. 33) is presently most commonly used.

## 3 Results

### 3.1 Introduction

We performed DFT calculations for 34 models of phosphorylated amino acids in a variety of molecular environments. Aspartyl phosphate was represented by acetyl phosphate, serine or threonine phosphate by methyl phosphate, and tyrosine phosphate by *p*-tolyl phosphate as shown in Fig. 1. Different environments – generally asymmetric – were modeled by interactions with either H_2_O or HF molecules. The aim was to correlate vibrational wavenumbers with structural properties. As in our previous work,^19^ we did not see a different behavior of the H_2_O and HF environments as they both follow the same trends in the correlations. The same is true for the *ε* = 4 and *ε* = 80 environments and both conclusions are supported by calculations for 48 model structures with the smaller basis set 6-31++G**. However, this basis set performed considerably worse in wavenumber calculations^25^ and is therefore not considered here.

### 3.2 Correlations between the vibrational spectrum and P–O bond lengths

Table 1 gives an overview of the quality of correlations between bond lengths and P–O wavenumbers. The best correlation (*i.e.* with the largest coefficient of determination *R*^2^) is that between the highest *ṽ*_as_(P–O_T_) wavenumber and the bond length of the shortest P–O_T_ bond. Also the mean P–O_T_ bond length can be well predicted with *R*^2^ values close to 0.9, either from the fundamental wavenumber or from the mean wavenumber of the asymmetric P–O_T_ stretching vibrations. Other useful correlations are those between the P–O_B_ bond length and the mean asymmetric wavenumber and between the largest P–O_T_ bond length difference and the wavenumber difference Δ*ṽ*_as_(P–O_T_). The longest P–O_T_ bond is predicted considerably less well with an *R*^2^ value of only 0.55.

**Table 1:** Correlations between phosphate bond lengths and vibrational spectrum. *R*^2^ is the coefficient of determination, min, mean and max indicate smallest, average and largest bond length or wavenumber. *ṽ*_as_ indicates the wavenumber of an asymmetric P–O_T_ stretching vibration, Δ*ṽ*_as_(P–O_T_) is the wavenumber difference between the two asymmetric stretching vibrations and Δ(P–O_T_) the difference between the shortest and longest P–O_T_ bond in a given structure. *ṽ*_f_ is the fundamental wavenumber^18^ defined as *ṽ*_f_ = [(*ṽ*_as1_^2^ + *ṽ*_as2_^2^ + *ṽ*_s_^2^)/3]^1/2^, where *ṽ*_as1_ and *ṽ*_as2_ are the wavenumbers of the two asymmetric stretching vibrations and *ṽ*_s_ the wavenumber of the symmetric stretching vibration. The best correlations are indicated by *R*^2^ values in bold print

Bond	*R* ^2^ for correlation with				
	Min *ṽ*_as_(P–O_T_)	Mean *ṽ*_as_(P–O_T_)	Max *ṽ*_as_(P–O_T_)	*ṽ*_f_	Δ*ṽ*_as_(P–O_T_)
P–O_B_	0.55	**0.79**	0.53	0.66	0.00
Min P–O_T_	0.06	0.56	**0.94**	0.58	0.5
Mean P–O_T_	0.50	0.88	0.72	**0.90**	0.04
Max P–O_T_	**0.55**	0.35	0.07	0.36	0.15
Δ(P–O_T_)	0.14	0.03	0.40	0.04	**0.81**

The C–O_B_ bond length does not correlate with any of the vibrational properties considered in Table 1. Instead, the data points cluster according to the nature of the molecules. This lack of relationship might be expected, since the P–O_T_ asymmetric stretching vibrations are good group vibrations with very little contribution from the C–O_B_ stretching vibration.

There is no spectroscopic parameter that provides acceptable correlations with all P–O bond properties studied in Table 1. Instead, a specific spectroscopic parameter has to be used for each of the structural parameters. The fundamental wavenumber^18^ (see heading of Table 1), which was proposed to be a geometry independent parameter, provides indeed the best correlation with the mean P–O_T_ bond length. The *R*^2^ values of this and of other correlations with the fundamental wavenumber are often very similar to those with the mean wavenumber of the asymmetric stretching vibrations.

No correlations were found between the symmetric P–O_T_ stretching vibration and the bond parameters considered in Table 1. Instead the data points form clusters for each of the studied molecules. The likely reason for this observation is that the symmetric stretching vibration is less localized on the phosphate group than the asymmetric stretching vibrations.

**Fig. 2:**
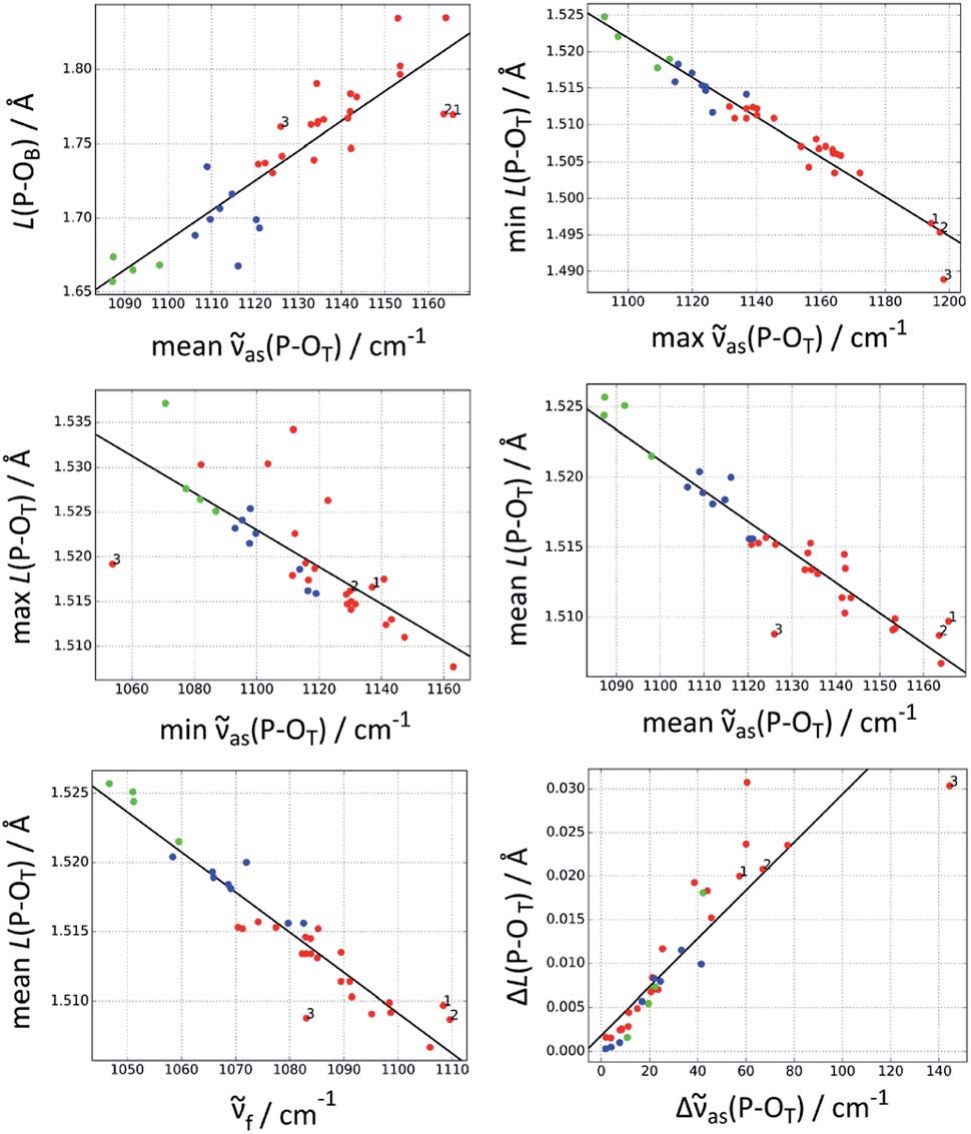
The best correlations between P–O_T_ stretching vibrations and P–O bond lengths *L*(P–O). Red: acetyl phosphate, green: methyl phosphate, blue: *p*-tolyl phosphate. The three acetyl phosphate models for the catalytic site of the Ca^2+^-ATPase are numbered (see Discussion). For further explanations, see the heading of Table 1.

Fig. 2 shows the best correlations, Table 2 lists additional parameters for these and Table 3 the parameters for the trend lines. The standard deviation of the bond length parameters from the trend line (Table 2) is ≤0.5 pm for the P–O_T_ bonds and 2 pm for the P–O_B_ bond. The standard deviation is ∼10% of the span of bond lengths in the data set for most correlations, it is 5% for the best correlation, which predicts the shortest P–O_T_ bond.

**Table 2:** Properties of the best correlations between phosphate bond lengths and wavenumber of vibration. *L* range is the range of bond lengths found in our models, Δ*L* the difference between the longest and shortest bond of our models, STD(*L*) is the standard deviation of the bond length values from the trend line, STD(*L*)/Δ*L* is a measure of the quality of the prediction, vibration is the vibration used for the correlation. See Table 1 for further explanations

Bond	*L* range/Å	STD(*L*)/Å	STD(*L*)/Δ*L*	Vibration
P–O_B_	1.657–1.835	2 × 10^−2^	0.12	Mean *ṽ*_as_(P–O_T_)
Min P–O_T_	1.489–1.525	2 × 10^−3^	0.05	Max *ṽ*_as_(P–O_T_)
Mean P–O_T_	1.509–1.526	2 × 10^−3^	0.10	*ṽ*_f_
		2 × 10^−3^	0.10	Mean *ṽ*_as_(P–O_T_)
Max P–O_T_	1.511–1.537	5 × 10^−3^	0.17	Min *ṽ*_as_(P–O_T_)
Δ(P–O_T_)	0.000–0.031	4 × 10^−3^	0.12	Δ*ṽ*_as_(P–O_T_)

**Table 3:** Linear correlations between *ṽ*_as_(P–O_T_) wavenumbers and P–O bond lengths. The data were fitted with a line *L* = *aṽ* + *b*. Where *L* is the bond length in Å, *ṽ* the wavenumber in cm^−1^, *a* the slope in Å cm and *b* the intercept in Å. The correlations are applicable in the intervals given in Table 2 for each bond. See Table 1 for further explanations

Bond	*a*/Å cm	*b*/Å	P–O_T_ vibration used for the correlation
P–O_B_	2.00 × 10^−3^	−0.52	Mean *ṽ*_as_(P–O_T_)
Min P–O_T_	−2.71 × 10^−4^	1.820	Max *ṽ*_as_(P–O_T_)
Mean P–O_T_	−2.90 × 10^−4^	1.828	*ṽ*_f_
	−2.18 × 10^−4^	1.761	Mean *ṽ*_as_(P–O_T_)
Max P–O_T_	−2.07 × 10^−4^	1.750	Min *ṽ*_as_(P–O_T_)
Δ(P–O_T_)	2.76 × 10^−4^	2 × 10^−3^	Δ*ṽ*_as_(P–O_T_)

### 3.3 The nature of the high wavenumber asymmetric stretching vibration

**Fig. 3:**
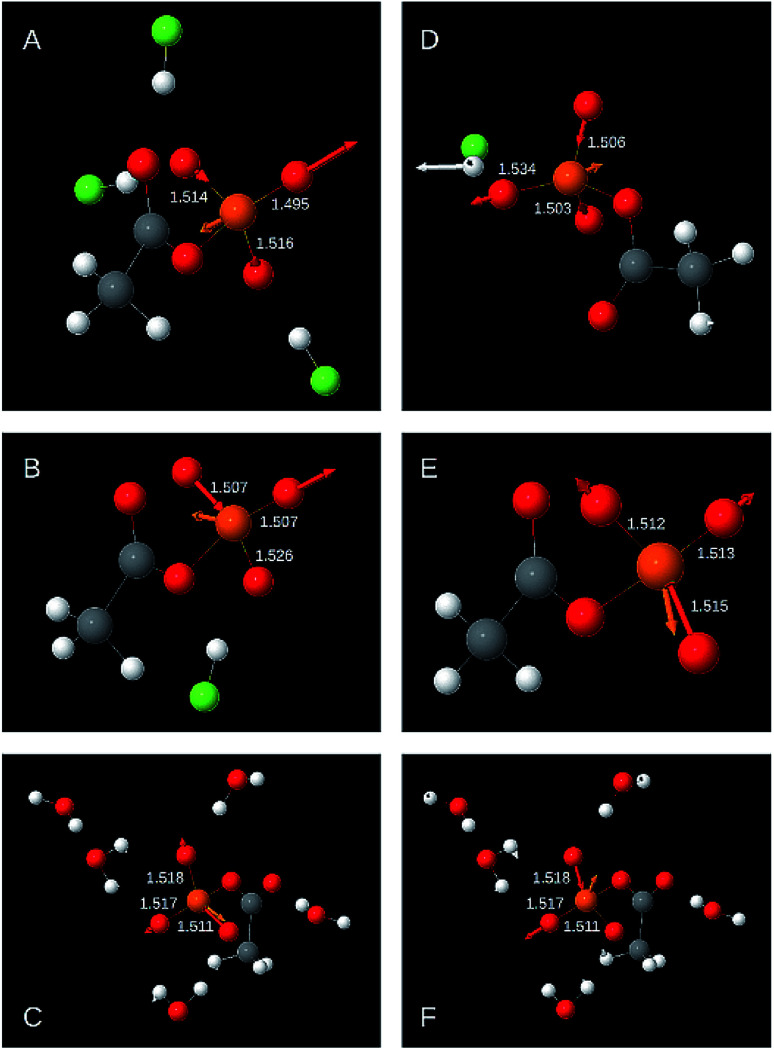
Displacement vectors associated with the asymmetric P–O_T_ stretching vibrations for selected AcP models. (A)–(C) High wavenumber vibration max *ṽ*_as_(P–O_T_). (D)–(E) Low wavenumber vibration min *ṽ*_as_(P–O_T_). (A) Example for a strong contribution of the shortest P–O_T_ bond. The energy contributions of the shortest, middle, and longest bond were 74%, 16%, and 11% respectively. Contribution refers to the relative energy contribution of a particular P–O_T_ bond to the total contribution of all P–O_T_ bonds. The contributions do not add up to 100% because of rounding errors. (B) Example for a strong contribution of the middle P–O_T_ bond and relatively small contributions of the shortest and the longest P–O_T_ bonds (energy contributions 50%, 50%, 0%). (C) Example for a relatively strong contribution of the longest P–O_T_ bond (energy contributions 69%, 15%, 15%). (D) Example for a relatively strong contribution of the shortest bond and a relatively small contribution of the longest P–O_T_ bond (energy contributions 21%, 32%, 47%). (E) Example for a strong contribution of the longest P–O_T_ bond (energy contributions 14%, 20%, 66%). (F) Example for a strong contribution of the middle P–O_T_ bond and a weak contribution of the shortest bond (energy contributions 0%, 49%, 51%). The structural model is the same as in panel C. The figure was generated with Jmol.

The following two sections discuss the relative energy contributions of the P–O_T_ bonds to the asymmetric stretching vibrations. A value of 100% refers to the contribution of all three bonds together. The energy contribution of the P–O_B_ bond to these two vibrations is negligible (≤0.5%). The atomic movements associated with the two asymmetric P–O_T_ stretching vibration are shown in Fig. 3 for selected structural models. Panels A–C refer to the high wavenumber vibration and panels D–F to the low wavenumber vibration.

As shown in Fig. 2 and Table 1, the high wavenumber asymmetric vibration correlates best with the shortest P–O_T_ bond length (*L*_s_). This is not surprising as this bond contributes most to the energy of this vibration (40–80% of the total contribution of all P–O_T_ stretching vibrations). Its contribution is high (70–80%) when there is a considerable difference between the bond lengths of the two shortest bonds (>0.01 Å), but it can be nearly equally high when there is little bond length difference between the three P–O_T_ bonds. There is also a weak correlation between the energy contribution and the bond length difference between shortest and middle bond Δ*L*_ms_ = *L*_m_ − *L*_s_ normalised to the difference between the shortest and the longest bond Δ*L*_ls_ = *L*_l_ − *L*_s_: the energy contribution of the shortest bond increases with increasing Δ*L*_ms_/Δ*L*_ls_. Such normalised bond length differences will be named relative bond length differences in the following.

The middle P–O_T_ bond contributes 10 to 60%. When there is a considerable bond length difference between the middle and the longest bond (>0.01 Å), the shortest and the middle bond contributions are nearly equal. Weak trends indicate that the middle P–O_T_ bond contributes more to the high wavenumber asymmetric stretching vibration when the relative bond length difference with the longest bond (Δ*L*_lm_/Δ*L*_ls_) increases and that with the shortest bond (Δ*L*_ms_/Δ*L*_ls_) decreases.

The longest bond contributes 0 to 16% (in one model 24%). When the bond lengths of longest and middle bond are considerably different (>0.01 Å), the contribution from the longest bond is close to zero.

In most models (31 out of 34), the two longer bonds vibrate in phase and out of phase with the shortest bond. Only when the energy contribution of the longest bond is less than 1% of the energy contribution of all P–O_T_ bonds, the longest bond might vibrate in phase with the shortest bond.

### 3.4 The nature of the low wavenumber asymmetric stretching vibration

The shortest bond contributes 0–35% to the asymmetric stretching vibration with lower wavenumber. It contributes most when its bond length is similar to that of the middle bond. Its contribution is below 5% when the bond length difference between the shortest and the middle bond is considerable (≥0.01 Å).

The middle bond contributes 13–55%. When the bond length difference between shortest and middle bond Δ*L*_ms_ is above 0.01 Å, large contributions were obtained. Below 0.01 Å both large and small contributions were calculated.

The longest bond contributes 45 to 70%. Only in one case the contribution was less (31%). There is no obvious correlation between the bond length differences Δ*L*_ms_ and Δ*L*_lm_ and the energy contribution of the longest bond.

In the low wavenumber asymmetric stretching vibration, the longest bond moves out of phase with the middle bond and generally also out of phase with the shortest bond. Only in two out of 34 models, the longest bond moves in phase with the shortest bond and out of phase with the middle bond. In these cases, the shortest bond hardly moves and its energy contribution to the total contribution of all P–O_T_ bonds is less than 1%.

### 3.5 Correlations between vibrational spectrum and phosphate bond angles

We investigated also correlations between vibrational spectrum and phosphate bond angles. Their inspection shows that bond angles correlate less with the asymmetric P–O_T_ stretching vibrations than the P–O bond length parameters discussed so far. The *R*^2^ values of the best bond angle correlations are between 0.61 and 0.66 but strong outliers make these correlations less valuable for analytical purposes. Bond angle correlations are shown and discussed in the ESI.†

## 4 Discussion

### 4.1 Determination of P–O_T_ bond lengths from the vibrational spectrum

A large number of correlations exist between vibrational spectra and chemical properties of compounds.^34–36^ This work deals with structural properties of phosphate groups and extends the work by Deng *et al.*^18^ to models for phosphorylated amino acids in asymmetric environments. Deng *et al.* related the wavenumbers of the P–O_T_ stretching vibrations to the bond valence^31^ of the P–O_T_ bonds, which in turn can be used to calculate their bond length. Here, we established a number of direct correlations between DFT calculated wavenumbers and structural parameters of the phosphate group. The accuracy of these correlations cannot be better than the accuracy of the DFT calculations. Therefore, we used a large basis set that reproduces well the experimental wavenumbers.^25^ The accuracy of bond length calculations with DFT is discussed below.

Our best correlations are between the mean P–O_T_ bond length and the fundamental or the mean asymmetric wavenumber, as well as between the shortest P–O_T_ bond length and the highest asymmetric wavenumber, with standard deviations from the trend line of only 0.002 Å (0.2 pm).

The following section gives an example of how these correlations can be beneficially used to analyze the catalytic site of proteins. It relates to our previous work on the Ca^2+^-ATPase^16^ in which we studied the E2P phosphoenzyme intermediate of this enzyme. An isotope exchange experiment was used to identify the phosphate vibrations in the infrared spectrum. We found that the *ṽ*_as_(P–O_T_) vibrations in the ATPase environment (1194, 1137 cm^−1^) were upshifted compared to an aqueous environment (two degenerate vibrations at 1132 cm^−1^). These wavenumbers will now be used in combination with our correlations to analyze the structure of the E2P phosphoenzyme intermediate.

The shortest P–O_T_ bond of the Ca^2+^-ATPase E2P phosphoenzyme is predicted to be 1.496 Å long (using the correlation with the highest *ṽ*_as_(P–O_T_) wavenumber). AcP-E2P model 1, which reproduces the experimental *ṽ*_as_(P–O_T_) wavenumbers of E2P, lies on the trendline. The correlation between the splitting of the two asymmetric wavenumbers and the maximum bond length difference predicts the longest bond to be 0.018 Å longer than the shortest. In AcP-E2P model 1 it is 0.020 Å longer. From these two correlations, the longest bond is therefore expected to be 1.514 Å (=1.496 Å + 0.018 Å), the correlation between longest bond and lower *ṽ*_as_(P–O_T_) predicts 1.515 Å and AcP-E2P model 1 gives 1.517 Å, which is in good agreement. Using the correlation with the mean P–O_T_ bond length, this length is predicted to be 1.507 Å, which gives a middle P–O_T_ bond length of 1.511 Å (=3 × 1.507 Å − 1.496 Å − 1.514 Å). In AcP-E2P model 1 this bond is 1.516 Å long. In conclusion, the E2P phosphate group is predicted to have one P–O_T_ bond that is considerably shorter than the other two. This is in agreement with our DFT calculations of ∼150 atom models of the ATPase catalytic site, but at a lower level of theory, where the shortest bond is 0.02 and 0.03 Å shorter than the other two.^8^ Thus, the trends seen in the small molecule models are reflected in the larger, but less accurate models of the catalytic site.

The bond lengths for the E2P phosphoenzyme can now be compared to an aqueous environment. The experimental wavenumber of the degenerate asymmetric vibration of AcP in water is 1132 cm^−1^,^16^ which gives a shortest bond length of 1.513 Å. This number is in excellent agreement with the average value of the five AcP models that closely reproduce the experimental wavenumbers in water (1.512 Å). The other P–O_T_ bonds are of similar length, as the mean P–O_T_ bond length is predicted from the correlation to be 1.514 Å, which is similar to the average bond length of the AcP water models of 1.513 Å. A comparison of these values to those derived for the E2P phosphoenzyme shows that the two longer P–O_T_ bonds of E2P have a similar length as those in water and therefore their oxygens experience interactions of similar strength as in an aqueous environment. However, the shortest ATPase bond is predicted to be ∼0.02 Å shorter than the shortest bond in aqueous environment, which indicates weaker interactions of this terminal oxygen atom with the protein environment than in water.

### 4.2 Determination of the P–O_B_ bond length from the vibrational spectrum

Probably the most interesting of our correlations is that between the mean asymmetric wavenumber and the P–O_B_ bond length. This is the bond that links the phosphate group to amino acid side chains in phosphorylated proteins and is the one that is cleaved in enzymatic reactions. The correlation makes it possible to assess the bond strength of the scissile P–O_B_ bond and thus whether an enzyme weakens this bond already in the ground state prior to the dephosphorylation step in order to facilitate bond cleavage.

At first sight it might seem surprising that there is a correlation between the vibrations of the P–O_T_ bonds and the length of the P–O_B_ bond because the energy contribution of the latter to the P–O_T_ stretching vibrations is negligible. However, the bonds are related *via* the bond valence model,^30,31^ which states that the sum of the bond valences around a phosphorus atom should be 5 valence units. Thus, when the bond valences of the P–O_T_ bonds are known, that of the P–O_B_ bond can be calculated and this makes it also possible to calculate its bond length.

In the work of Deng *et al.*,^18^ the bond valence model was used for the evaluation of phosphate vibrations. They introduced the fundamental wavenumber (originally frequency) as spectroscopic parameter that is independent of phosphate geometry. Indeed, it is the best predictor for the mean P–O_T_ bond length. Nevertheless, the P–O_B_ bond is better predicted by the mean asymmetric wavenumber. This work shows that there is no single spectroscopic parameter that provides good correlations with all structural properties of the phosphate group. Instead, a specific spectroscopic parameter should be used for each structural parameter.

### 4.3 Outliers in the P–O_B_ bond length *versus* wavenumber correlation

The P–O_B_ bond was also in the focus of our previous work on the Ca^2+^-ATPase.^16^ Using the approach of Deng *et al.*,^18^ we concluded erroneously that the E2P ground state weakens the P–O_B_ bond. This error was revealed when we performed DFT calculations on models of the E2P catalytic site.^8^

The present study shows that there is indeed a correlation between P–O_T_ wavenumbers and P–O_B_ bond length, which was the basis of our original interpretation.^16^ When the mean asymmetric (or fundamental) wavenumber increases, the bond length also increases. However, the present study also shows that there are considerable deviations from the trend line. In particular, AcP-E2P models 1 and 2 are outliers in the correlation shown in Fig. 2 (top left panel) which are located below the trend line indicating that their P–O_B_ bonds are shorter than expected from their P–O_T_ wavenumbers.

In contrast, those acetyl phosphate models that reproduced the experimental wavenumbers of acetyl phosphate in water lie on the trend line. Therefore, they have lower wavenumbers than the AcP-E2P models, but a similar P–O_B_ bond length.

One reason for the deviation of AcP-E2P models 1 and 2 from the trend line might be an asymmetric environment and such an asymmetric environment is indicated by the wavenumber splitting of the two asymmetric stretching vibrations. However, four structural models with a similar or a larger splitting than AcP-E2P models 1 and 2 are close to the trend line, indicating that an asymmetric environment cannot explain the deviation of AcP-E2P models 1 and 2 from the trend line.

A further reason might be that the P–O_T_ wavenumbers do not faithfully reflect the P–O_T_ bond lengths and bond valences, and therefore cannot be used to predict the P–O_B_ bond length. Indeed, AcP-E2P models 1 and 2 have a slightly larger mean P–O_T_ bond length than predicted by the mean asymmetric wavenumber (middle right panel of Fig. 2) and a lower mean bond valence (not shown). This results in a larger bond valence for the P–O_B_ bond than expected and therefore a shorter P–O_B_ bond. However, the deviation of the AcP-E2P models from the trend line is relatively week, in particular for AcP-E2P model 2, and therefore the discussed deficiency is likely not the main reason for the difficulty to predict the P–O_B_ bond length from the mean asymmetric P–O_T_ wavenumber.

A third reason could be that the bond valence model fails to predict the P–O_B_ bond lengths from the P–O_T_ bond lengths. To check this, we used the DFT P–O_T_ bond lengths to calculate the P–O_T_ bond valences, then the P–O_B_ bond valence and finally the P–O_B_ bond length. We thus obtained a plot of the DFT P–O_B_ bond length against the P–O_B_ bond length predicted from the bond valence model (not shown) which was very similar to the plot of the DFT P–O_B_ bond length against the mean asymmetric wavenumber (top left panel of Fig. 2). In particular, AcP-E2P models 1 and 2 were outliers in this plot too and located below the trend line. Their predicted P–O_B_ bonds were longer than those of the acetyl phosphate models for an aqueous environment, whereas their DFT P–O_B_ bond lengths were similar. The plot indicates that most outliers in the top left panel of Fig. 2 are due a failure of the bond valence model to calculate the P–O_B_ bond length from the DFT P–O_T_ bond lengths.

For outliers above the trend line in Fig. 2 an explanation for their deviating behavior could be found. The respective models have an interaction between the phosphorus atom and the oxygen or fluorine atom of a ligand. This results in a pentacoordinated phosphorus with a certain amount of bond valence in the bond between the phosphorus and the attacking nucleophile. The bond valence of all five bonds is expected to sum up to a value of 5 valence units, which implies that the bond valence sum of the four P–O bonds is less than 5 valence units. Indeed, the models above the trend line have all P–O bond valence sums at the lower end of the bond valence sum range calculated for our model structures. As a consequence, their P–O_B_ bond lengths cannot be predicted accurately from the P–O_T_ bond lengths: two structures with the same P–O_T_ bond lengths and thus the same P–O_T_ wavenumbers will have different P–O_B_ bond lengths depending on whether a nucleophile is close to the phosphorus atom or not. With nucleophile, the bond valence of the P–O_B_ bond is smaller than without (because the bond valence that is not accounted for by the P–O_T_ bonds is distributed over two bonds) and the P–O_B_ bond therefore longer. This makes models with a phosphorus–nucleophile interaction lie above the trend line in the top left panel of Fig. 2.

Models that appear considerably below the trend line, as AcP-E2P models 1 and 2 all have a bond valence sum at the higher end of the bond valence sum range. However, we did not find an obvious molecular interpretation for this observation.

### 4.4 Accuracy of DFT geometry calculations

As mentioned above, the sum of bond valences of the P–O bonds can be expected to be equal to the atomic valence of phosphorus, 5 valence units, for the thirty models without an interaction of the phosphorus with an oxygen or fluorine atom. However, the P–O bond valence sums calculated from the DFT bond lengths were below 5 also for these models. They were in the range of 4.52–4.77 valence units calculated with the two relationships between bond valence and bond lengths and with different parameters for P–O bonds (see Table 4). The highest bond valence sum is obtained for eqn (1) with the parameters of Brown and Wu 1976^37^ and the lowest sum for eqn (2) and the parameters 1.604 and 0.37 for *L*_1_ and *B*, respectively.^38^ Also Launay *et al.*^39^ find a bond valence sum in this range for phosphate, 4.63 valence units, in their DFT calculations because their calculated P–O bond lengths are ∼1% or ∼0.015 Å longer than experimentally observed.

**Table 4:** Correction of DFT bond lengths required to comply with the bond valence model. DFT bond lengths were either changed by a fixed amount (reported as change in Å) or by multiplication with a factor (reported as % change in brackets). The deviation of the sum of the bond valences of all four P–O bonds from the expected value of 5 was minimized. The column “Parameters” states the bond valence equation and *L*_1_ and *N* for eqn (1) and *L*_1_ and *B* for eqn (2)

Parameters	Change of DFT bond length required to minimize P–O bond valence sum deviation from 5
Eqn (1): 1.622 Å, 4.290 Å (ref. 37)	−0.017 Å (−1.1%)
Eqn (2): 1.604 Å, 0.370 Å (ref. 38)	−0.038 Å (−2.4%)
Eqn (2): 1.615 Å, 0.370 Å (ref. 44)	−0.027 Å (−1.7%)
Eqn (2): 1.617 Å, 0.370 Å (ref. 33)	−0.025 Å (−1.6%)
Eqn (2): 1.624 Å, 0.399 Å (ref. 43)	−0.025 Å (−1.6%)

Therefore, we will discuss next studies that assessed the performance of DFT in predicting bond lengths and used a similar level of theory as in the present study. The functional in these studies was also B3LYP and the basis set was 6-311+G**, which is close to our 6-311++G** basis set. These studies reported mean unsigned errors between calculated and measured bond lengths of 0.008 Å (ref. 40) and 0.056 Å.^41^ The DFT bond lengths are longer than those obtained from experiments^40^ and there is considerable sensitivity of the mean unsigned bond length error to the type of bond.^41^ Unfortunately, P–O bonds were not included in these studies.

Inclusion of diffuse functions for H in our 6-311++G** basis set is not expected to change these errors much, as there is very little influence when diffuse functions are included for non-hydrogen atoms (from 6-311G** to 6-311+G**)^40^ or when diffuse functions for H and non-hydrogen atoms are added to the smaller basis set 6-31G* (giving 6-31+G* and 6-31++G*).^42^

### 4.5 Correction of DFT bond lengths

According to the above benchmark studies, we tested what decrease in bond length is needed to bring the bond valence sum close to 5 either by adding a summand to the DFT bond lengths (in the following reported as change in Å) or by multiplying the DFT bond lengths with a factor (reported as % change in brackets). The required correction depends on the bond valence equation and on the parameters used as listed in Table 4. With eqn (1) the DFT bond lengths need to be shortened by 0.017 Å (1.1%) and with eqn (2) by 0.25–0.27 Å (1.6–1.7%) for most parameter sets. The largest correction is 0.038 Å (2.4%) for the parameter set by Brese and O'Keeffe, (1991).^38^ This parameter set performed worse for pentavalent phosphorus than the other sets in the study by Gagné and Hawthorne^43^ who used the most comprehensive data set for deriving bond valence data. The other sets performed similar, with the parameter set proposed by Gagné and Hawthorne performing best. Eqn (1) was not tested in that study. For the three best parameter sets, the root mean square deviation between the bond valence sum around phosphorus and its valence of 5 was ∼0.1 valence units in the study by Gagné and Hawthorne. For the corrected DFT bond lengths in our study, the respective root mean square deviations were 0.03 valence units for eqn (1) and 0.04 valence units for eqn (2) and all four parameter sets. We conclude that the deviations from the expected bond valence sum for our corrected DFT bond lengths are similar to those observed experimentally.

Using our results with the Gagné and Hawthorne parameters as a reference, we conclude that good agreement between the empirical bond valence model and our DFT calculations can be obtained when it is assumed that the DFT bond lengths are overestimated by ∼0.025 Å (2.5 pm) or 1.6%. This conclusion is in line with the above DFT benchmark studies.

We also tested how well the bond valence model predicts DFT P–O_B_ bond lengths from the DFT P–O_T_ bond lengths. All DFT bond lengths were corrected by a new correction summand or correction factor in order to minimize the deviation between the corrected DFT P–O_B_ bond length and the P–O_B_ bond length predicted by the bond valence model from the corrected DFT P–O_T_ bond lengths. For all five parameter sets, the optimal corrections turned out to be within 0.01% or 0.0001 Å of the corrections needed to bring the bond valence sum close to 5 (Table 4). Thus, the best prediction of the P–O_B_ bond length from the P–O_T_ bond lengths is obtained when the bond valence around phosphorus is 5, which demonstrates that bond valence model and DFT results are fully consistent after correction of the DFT bond lengths.

## 5 Conclusions

This work presents several useful correlations between structural properties of the phosphate group and its vibrational spectrum. They were obtained for model compounds of phosphorylated amino acids and are therefore applicable for the study of phosphorylated proteins. The best correlations have a standard deviation from the trend line of only 0.2 pm, which highlights the structural sensitivity of vibrational spectroscopy. They are a further demonstration “that the resolution of vibrational spectroscopy picks up where diffraction and multidimensional nuclear magnetic resonance (NMR) techniques leave off, at *ca.* 0.2 Å, and extends down to much lower lengths.”^35^

## Conflicts of interest

There are no conflicts to declare.

## Supplementary Material

RA-010-C9RA10366J-s001

RA-010-C9RA10366J-s002
