# Supplementary material for: Correlations between the structure and the vibrational spectrum of the phosphate group. Implications for the analysis of an important functional group in phosphoproteins
Source: RSC Adv. 2020 Jan 29;10(8):4715–24. doi: 10.1039/c9ra10366j (PMC9049017; doi:10.1039/c9ra10366j)
Supplement: RA-010-C9RA10366J-s001 [file RA-010-C9RA10366J-s001.pdf]

Electronic Supplementary Material (ESI) for RSC Advances.
This journal is © The Royal Society of Chemistry 2020

Electronic Supplementary Information

Correlations between the structure and the vibrational spectrum of the phosphate group. Implications for the analysis of an important functional group in phosphoproteins.

Pontus Pettersson, and Andreas Barth*

Department of Biochemistry and Biophysics, Stockholm University, Sweden

* corresponding author, email: barth@dbb.su.se

Correlations between vibrational spectrum and phosphate bond angles

Tables S1 to S3 list the properties of correlations between the O—P—O bond angles and the wavenumbers of the P—O_T stretching vibrations. As indicated by the R^2 values listed in Table S1, the O_B—P—O_T angles correlate best with the average wavenumber of the asymmetric vibrations, whereas two of the O_T—P—O_T angles correlate best with a specific asymmetric vibration. The fundamental wavenumber never provides the best correlation. The splitting of the two asymmetric P—O_T stretching vibrations does not correlate with any of the angles, the R^2 values are mostly below 0.1. No correlations exist between the P—O stretching vibrations and the C—O_B—P angle. A comparison with the R^2 values in Table 1 of the main text reveals that the bond angles are less well predicted than most of the P–O bond length parameters.

Table S1. Correlations between phosphate bond angles and vibrational spectrum. R^2 is the coefficient of determination, Min, Mean and Max indicate smallest, average and largest angle or wavenumber. $\tilde{\nu}_{as}$ indicates the wavenumber of an asymmetric P—O_T stretching vibration. $\tilde{\nu}_f$ is the fundamental wavenumber[18] defined as $\tilde{\nu}_f = [(\tilde{\nu}_{as1}^2+\tilde{\nu}_{as2}^2+\tilde{\nu}_s^2)/3]^{½}$, where $\tilde{\nu}_{as1}$ and $\tilde{\nu}_{as2}$ are the wavenumbers of the two asymmetric stretching vibrations and $\tilde{\nu}_s$ the wavenumber of the symmetric stretching vibration. The best correlations are indicated by R^2 values in bold print.

**Angle**	**R^2 for correlation with**			
	**Min $\tilde{\nu}_{as}$(P—O_T)**	**Mean $\tilde{\nu}_{as}$(P—O_T)**	**Max $\tilde{\nu}_{as}$(P—O_T)**	**$\tilde{\nu}_f$**
Min O_B—P—O_T	0.24	**0.51**	0.47	0.41
Mean O_B—P—O_T	0.36	**0.61**	0.48	0.46
Max O_B—P—O_T	0.27	**0.33**	0.19	0.21
Min O_T—P—O_T	**0.63**	0.53	0.19	0.39
Mean O_T—P—O_T	0.38	**0.64**	0.50	0.48
Max O_T—P—O_T	0.16	0.56	**0.66**	0.44

Figure S1 shows the best correlations between angles and P—O_T wavenumbers, Table S2 provides additional properties and Table S3 lists their parameters for the fitted linear equations. According to Table S2, the standard deviation of the bond angles from the trend line is smaller for the O_T—P—O_T angles than for the O_B—P—O_T angles. Of the latter, the mean O_B—P—O_T angle has the lowest standard deviation and the highest R^2.

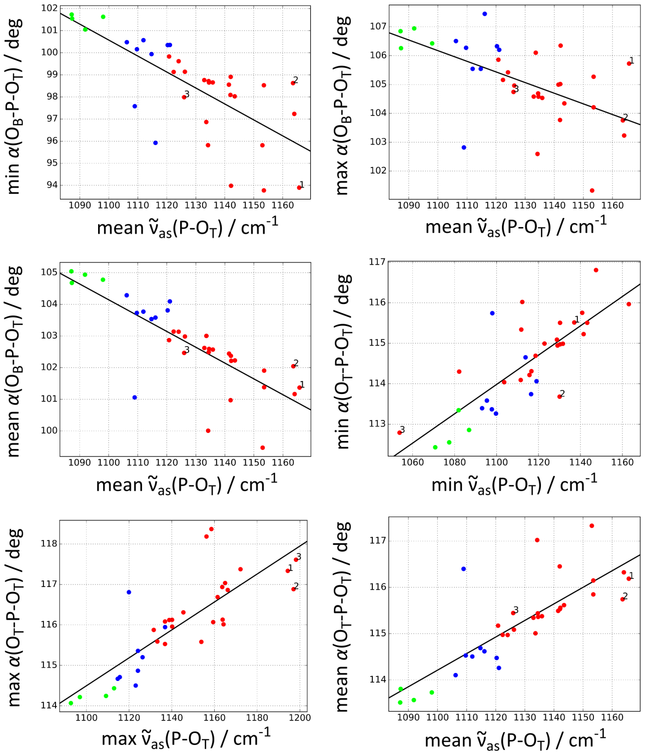

Figure S1. The best correlations between P—O_T stretching vibrations and O—P—O angles. Red: acetyl phosphate, green: methyl phosphate, blue: p-tolyl phosphate. The three acetyle phosphate models for the catalytic site of the Ca^{2+}-ATPase are numbered (see *Discussion* of the main text). For further explanations, see the heading of Table S1.

Table S2. Correlations between bond angles and wavenumber of vibration. α range is the range of bond angles found in our models, $\Delta\alpha$ is the difference between the largest and the smallest angle in the models, $STD(\alpha)$ is the standard deviation of the bond angle values from the trend line, $STD(\alpha)/\Delta\alpha$ is a measure of the quality of the prediction, vibration is the vibration used for the correlation. See Table S1 for further explanations.

**Bond angle**	**α range / deg**	**$STD(\alpha)$ / deg**	**$STD(\alpha)$ / $\Delta\alpha$**	**Vibration**
Min O_B—P—O_T	93.77-101.73	1.5	0.19	Mean $\tilde{\nu}_{as}$(P—O_T)
Mean O_B—P—O_T	99.47-105.04	0.8	0.15	Mean $\tilde{\nu}_{as}$(P—O_T)
Max O_B—P—O_T	101.33-107.45	1.1	0.18	Mean $\tilde{\nu}_{as}$(P—O_T)
Min O_T—P—O_T	112.43-116.81	0.7	0.15	Min $\tilde{\nu}_{as}$(P—O_T)
Mean O_T—P—O_T	113.51-117.33	0.6	0.15	Mean $\tilde{\nu}_{as}$(P—O_T)
Max O_T—P—O_T	114.07-118.37	0.7	0.15	Max $\tilde{\nu}_{as}$(P—O_T)

Table S3. Linear correlations between wavenumber and bond angle. The data were fitted with a line $\alpha = c\tilde{\nu}+d$. where α is the bond angle in deg, $\tilde{\nu}$ the wavenumber in cm^{-1}, c the slope in deg·cm and d the intercept in deg. The correlations are applicable in the intervals given in Table S2 for each bond angle.

**Bond angle**	***c* / deg cm**	***d* / deg**	**Vibration**
Min O_B—P—O_T	-7.22×10^{-2}	180.0	Mean $\tilde{\nu}_{as}$(P—O_T)
Mean O_B—P—O_T	-5.00×10^{-2}	159.1	Mean $\tilde{\nu}_{as}$(P—O_T)
Max O_B—P—O_T	-3.69×10^{-2}	146.8	Mean $\tilde{\nu}_{as}$(P—O_T)
Min O_T—P—O_T	3.62×10^{-2}	74.1	Min $\tilde{\nu}_{as}$(P—O_T)
Mean O_T—P—O_T	3.58×10^{-2}	74.9	Mean $\tilde{\nu}_{as}$(P—O_T)
Max O_T—P—O_T	3.45×10^{-2}	76.5	Max $\tilde{\nu}_{as}$(P—O_T)
